# Preoperative C-Reactive Protein-to-Albumin Ratio Predicts Postoperative Pancreatic Fistula following Pancreatoduodenectomy: A Single-Center, Retrospective Study

**DOI:** 10.3390/curroncol29120775

**Published:** 2022-12-14

**Authors:** Naotake Funamizu, Takeshi Utsunomiya, Masahiko Honjo, Chihiro Ito, Mikiya Shine, Mio Uraoka, Tomoyuki Nagaoka, Kei Tamura, Katsunori Sakamoto, Kohei Ogawa, Yasutsugu Takada

**Affiliations:** Department of Hepatobiliary Pancreatic and Transplantation Surgery, Graduate School of Medicine, Ehime University, 454 Shitsukawa, Toon-City 791-0295, Ehime, Japan

**Keywords:** C-reactive protein to albumin ratio, postoperative complications, postoperative pancreatic fistula

## Abstract

Postoperative pancreatic fistula (POPF) following pancreatoduodenectomy (PD) is a potentially lethal complication, and it is clinically important to determine its risk preoperatively. Although C-reactive protein-to-albumin ratio (CAR) is reported to be a prognostic marker for postoperative complications in several cancers, no evidence is currently available regarding the association between preoperative CAR and POPF following PD for periampullary tumors. This study examined whether preoperative CAR could predict POPF following PD. Clinical data were retrospectively retrieved from Ehime University Hospital. The optimal cut-off value for CAR was determined using receiver operating characteristic (ROC) curve analysis. This study enrolled 203 consecutive patients undergoing PD for periampullary tumors. The CAR value was significantly higher in the POPF group than in the non-POPF group (*p* < 0.001). According to the ROC curve analysis, the optimal cut-off value for CAR was 0.09. Patients with CAR ≥ 0.09 had higher incidence rates of POPF than their counterparts. CAR ≥ 0.09 was a risk factor for POPF in the multivariate logistic regression analysis (odds ratio 34.5, 95% confidence interval 11.75–101.38, *p* < 0.001). This is the first report demonstrating an association between CAR and POPF following PD. Preoperative CAR is an independent predictive marker for POPF following PD.

## 1. Introduction

Pancreatoduodenectomy (PD) is the standard surgical procedure for treating periampullary tumors. Although surgical techniques and perioperative management have drastically improved, PD-related postoperative complication (POC) rates still reach up to 54% [1]. Among the potential POCs, the most potentially fatal POC is postoperative pancreatic fistula (POPF). Additionally, POPF potentially causes wound infections, intraabdominal abscesses, and intraabdominal hemorrhage [2]. Moreover, recent evidence has shown that POPF unfavorably affects the prognosis in patients with pancreatic cancer [3]. We previously showed that the geriatric nutritional risk index (GNRI) could predict POCs among patients who underwent pancreatic resection for periampullary diseases [4,5,6,7]. Instead of GNRI as a nutritional parameter, we explored another novel indicator for the risk of POPF following PD. Kubo et al. [8] revealed that a higher preoperative C-reactive protein (CRP) level could lead to surgical site infection in patients with colorectal cancer. In contrast, a lower preoperative albumin level can increase the risk of POCs [9,10]. Thus, we considered the CRP-to-albumin ratio (CAR) as a novel parameter for predicting POPF following open PD. CAR represents not only the inflammatory status but also the nutritional status of patients and is simply calculated from the CRP value divided by the albumin value. Recent evidence has shown that CAR is a potential prognostic predictor for several types of cancers [11,12,13]. CAR is easily accessible and inexpensive, requiring only routine preoperative laboratory data, such as GNRI. However, there have been no studies on the association between preoperative CAR and POCs, including POPF. Thus, this study aimed to determine whether preoperative CAR has a predictive value for POPF. Identifying predictive markers for POPF may be useful for identifying patients at high risk of POPF.

## 2. Materials and Methods

### 2.1. Patient Population

Between August 2009 and March 2022, all patients scheduled to undergo open PD for periampullary tumors, including pancreatic head cancer, distal bile cancer, ampullary cancer, duodenal cancer, neuroendocrine neoplasm, and intraductal papillary mucinous neoplasm, were enrolled in this study at the Department of Hepato-biliary-pancreatic and Transplantation Surgery, Ehime University Graduate School of Medicine, Japan. We included patients who underwent radical PD. The exclusion criteria were as follows: (1) patients who underwent exploratory laparotomy or bypass operation, (2) patients who underwent total pancreatectomy due to additional resection, and (3) patients who lacked information about preoperative CRP and albumin levels. Surgeons with substantial experience in pancreatic surgery performed all surgical procedures. POCs and POPF were defined and clarified according to the Clavien–Dindo [14] and International Study Group for Pancreatic Surgery (ISGPS) 2016 classifications [15]. In this study, grade B or C was defined as POPF. Clinical data were collected from inpatient and outpatient medical records. The Ethics Committee of Ehime University approved the study protocol in 2022 (approval number: 2204007) and followed the 2013 Declaration of Helsinki. The requirement for informed consent to use the patients’ medical information was waived owing to the retrospective nature of this study.

### 2.2. Collection of Clinical and Laboratory Data

Clinical data were retrieved regarding patient demographics (sex and age), anthropometric parameters (height, weight, and body mass index (BMI)), American Society of Anesthesiologists (ASA) physical status classification, comorbidities including diabetes mellitus, type of tumors, laboratory data such as preoperative CRP and albumin levels, and CAR as preoperative parameters. Additionally, intraoperative and postoperative parameters, such as operation time, estimated blood loss, presence of blood transfusion, POCs, and POPF were collected from individual medical records.

### 2.3. Definition of CAR

CAR was calculated using preoperative CRP and serum albumin values. These data were obtained before the surgical procedure and calculated using the following formula: CAR *=* CRP *(*mg/dL*)/serum albumin (*g/dL*)* [16].

### 2.4. Perioperative Management and Follow-Up Study

Preoperatively, all patients underwent routine blood tests, including CRP and serum albumin assessment, tumor marker analysis, and physical examination. Prophylactic antibiotics were administered through the peripheral vein before the induction of anesthesia. All participants with PD routinely received proton pump inhibitors. Ascites amylase values were measured from the drainage on postoperative days (PODs) 1, 2, 3, 5, and 7 until drain removal. The reconstruction method of PD was performed using Child’s method. All patients underwent pancreatojejunostomy anastomosis using Kakita or Blumgart methods. A drainage tube in the pancreatic duct was placed in all patients. Moreover, according to the operator’s judgment, one or two drain tubes were also placed in the abdominal cavity and removed between postoperative days 4 and 10. Additionally, dynamic computed tomography was performed to evaluate fluid collection before drainage tube decannulation. All patients were followed up, from hospital discharge to 6 months at least.

### 2.5. Statistical Analyses

Patient backgrounds are expressed as median and interquartile ranges for nonparametric distributions. Categorical data are expressed as numbers and percentages. In contrast, statistical significance was determined using the Mann–Whitney *U* test, Pearson’s χ^2^ test, or Fisher’s exact test for patients’ backgrounds and outcomes. A receiver operating characteristic (ROC) curve was calculated to identify the optimal CAR cut-off value for POPF using Youden’s index. Univariate and multivariable logistic regression models were used to analyze the potential predictive factors affecting POPF. The cut-off value for continuous variables was calculated using the respective ROC curve. The multivariate analysis further included potential factors identified in the univariate analysis (*p* < 0.05). Statistical analyses were performed using GraphPad Prism version 5.0 (GraphPad Software Inc., La Jolla, CA, USA) and Statistical Package for the Social Sciences (SPSS) (SPSS Inc., Chicago, IL, USA). *p* values <0.05 were considered statistically significant.

## 3. Results

### 3.1. CAR and Clinicopathological Features

**Figure 1:**
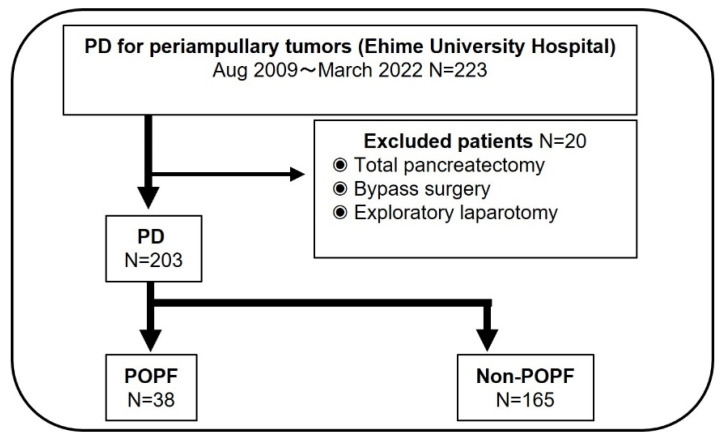
Study flow chart.

In this study, 223 patients underwent surgery for periampullary tumors, including pancreatic head cancer, distal bile cancer, ampullary cancer, duodenal cancer, neuroendocrine neoplasm, and intraductal papillary mucinous neoplasm during the same period. Except for 20 patients who met the exclusion criteria (total pancreatectomy, 1; bypass surgery, 12; exploratory laparotomy, 7; and lack of preoperative laboratory data, 0 patients), 203 patients were finally enrolled. The patients were divided into two groups according to the presence or absence of POPF: ISGPS classification ≥ grade B (Figure 1). In this study, POPF was observed in 38 of the 203 (18.7%) patients. The demographic and baseline characteristics of patients with and without POPF were compared (Table 1). There were no statistically significant differences in age, sex, ASA classification, rate of pancreatic and ampullary cancers, and presence of diabetes mellitus, excluding BMI and CAR, between the two groups (*p* = 0.042 and *p* < 0.001, respectively). Intraoperative variables, such as operation time (*p* = 0.003), estimated blood loss (*p* = 0.001), portal vein resection (*p* = 0.042), and soft pancreatic texture (*p* = 0.033) were statistically associated with the risk of POPF (Table 2).

### 3.2. Optimal CAR Cut-Off Value Measured by ROC Curve Analysis

**Figure 2:**
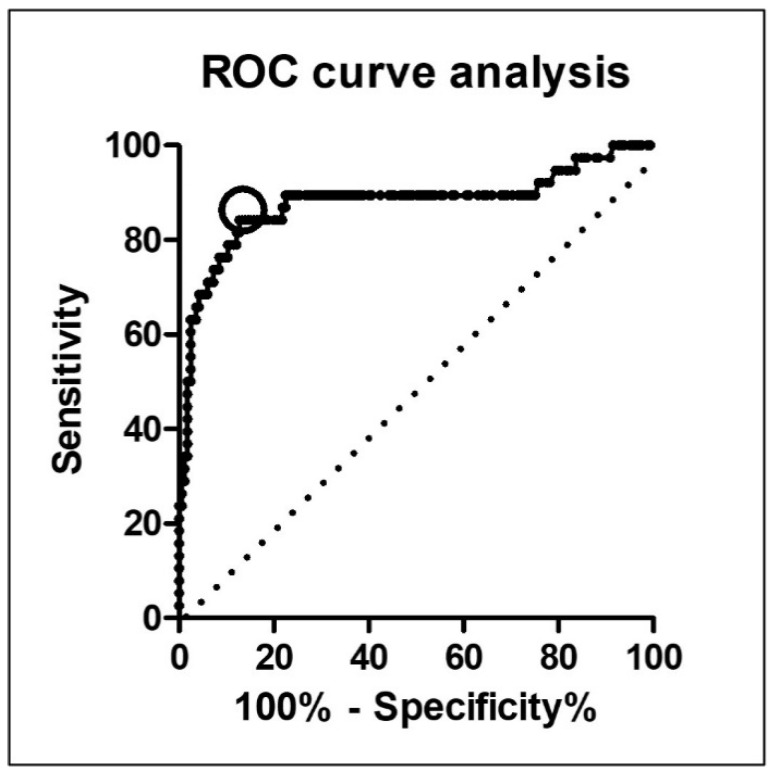
Optimal cut-off value selection for C-reactive protein-to-albumin ratio using receiver operating characteristic curve analysis.

The most appropriate cut-off value for evaluating the risk of POPF was determined using ROC curve analysis (Figure 2). With an area under the curve of 0.88 (95% confidence interval (CI), 0.80–0.96), the most appropriate cut-off value was 0.09. This value had a sensitivity of 84.2% and a specificity of 86.6%, with a likelihood ratio of 6.04. The incidence rate of POPF was significantly higher in patients with higher CAR (CAR ≥ 0.09, *N* = 55), and 58.2% (32/55) than in those with lower CAR (CAR < 0.09, *N* = 148) at 4.1% (6/148) (*p* < 0.001). Similarly, other cut-off values that were extracted by univariate analysis were similarly calculated using ROC curve analyses.

### 3.3. Multivariate Logistic Regression Analysis

Multivariate logistic regression analysis showed that CAR ≥ 0.09 (*p* < 0.001) and operation time ≥ 534 min (*p* = 0.018) were independent potential markers for POPF following PD, as outlined in Table 3.

## 4. Discussion

The rate of POC following PD remains high despite advanced surgical skills and perioperative management [1]. In addition, the mortality rate of PD ranges from 1.7% to 4.2% [17,18]. Rijssen et al. [3] showed that POPF was mainly attributable to worse mortality. Moreover, Linnemann et al. [1] showed that medical costs increased by about 50% after POCs following PD. Thus, adequate prevention or treatment of POPF can contribute to reducing the mortality rate. Accumulating evidence suggests that patient characteristics and perioperative conditions are associated with POPF following pancreatic resection, including higher BMI, soft pancreatic texture, lower serum albumin levels, higher CRP levels, and preoperative nutritional status [19,20,21]. However, a definitive risk factor for POPF has not yet been established. For such occasions, several POPF predictive scores, including inflammatory and/or nutritional status, have been developed, such as the GNRI [4,5], controlling nutritional status score [20], and prognostic nutritional index [22] to detect POPF at an earlier phase and with more precision. In addition, those scores using inflammatory or nutritional status, such as GNRI [23], the platelet-to-lymphocyte ratio [24], and neutrophil-to-lymphocyte ratio (NLR) [25,26], have been designed as prognostic factors in several types of cancers. Moreover, more recent reports have revealed that NLR, CRP-to-lymphocyte ratio (CLR), CAR, and GNRI play a critical predictive role in the incidence of POCs in pancreatic cancer [27,28]. Briefly, Huang et al. [27] revealed that NLR could predict POCs following PD . Fan et al. [28] showed that pretreatment CLR could be considered a feasible biomarker for the prognostic prediction of pancreatic cancer [28]. Actually, nutritional intervention with omega-3 fatty is a recommended strategy for pancreatic cancer patients to reduce POCs [29]. The common perception that the oncological characteristics and patient conditions influence the prognosis of patients with cancer has spread worldwide. In this manner, the clinical effect of novel scores, including nutrition and/or inflammatory indices, has been developed one after another to predict patient outcomes or postoperative complications.

Therefore, we evaluated whether the preoperative calculated CAR was associated with the risk of POPF following PD. Fairclough et al. [16] first reported that CAR, which includes CRP and albumin, is a predictive marker of mortality in acute medical admissions. Subsequently, several studies have consistently shown an association between CAR and cancer prognosis [30,31]. For example, Yoshida et al. [30] reported that CAR is associated with long-term outcomes of malignant pleural mesothelioma [30]. Recent evidence also revealed that CAR is a better prognostic factor in lung cancer [31]. Moreover, Zang et al.’s [32] meta-analysis also showed that CAR could be a useful prognostic biomarker in patients with pancreatic cancer who underwent surgery. Recently, Sakamoto et al. [33] showed that CAR on POD 3 is a reliable prediction marker of POPF following PD. This study was the first to prove the association between postoperative CAR and POPF. However, they confirmed the utility of postoperative calculated CAR values on POD3, which was the day for POPF diagnosis utilizing drain amylase level. We believe that preoperative availability is useful in patient risk evaluation and management of the perioperative period for surgeons to reduce the incidence of lethal conditions. Thus, no reports have focused on the association between higher preoperative CAR and the risk of POPF following PD. In this study, a preoperative CAR value of ≥ 0.09 was strongly correlated with the risk of POPF, supporting the clinical significance of preoperative nutritional assessment. This result suggests the clinical effect of nutritional assessment using preoperative CRP and albumin values. Poor preoperative nutritional condition or the presence of inflammation reflected by a lower CAR can affect postoperative recovery, including protracted wound healing. The precise mechanisms underlying the association between lower CAR and POPF should be determined in future studies. Finally, our study had a few limitations when interpreting the results. An important limitation was that this was a retrospective study with a relatively small sample size. Moreover, the data were collected from a single center. Therefore, a more comprehensive prospective study should be conducted in the future to validate our findings.

## 5. Conclusions

To the best of our knowledge, this is the first study investigating the association between preoperative CAR and POPF following PD. Although CAR is easily calculated using preoperative routine work without an invasive procedure and a high medical cost, it can predict the risk of POPF in patients following PD. 

## Figures and Tables

**Table 1:** Preoperative variables in patients with and without POPF.

Preoperative Variables	POPF Group	Non-POPF Group	*p*-Value
	*N* = 38)	(*N* = 165)	
Age (years)	73 (46–85)	71 (34–88)	0.435
Sex			
Male (%)	25 (65.8%)	94 (57.0%)	0.32
BMI	23.2 (16.5–29.4)	21.7 (14.0–32.1)	0.042
ASA classification			0.393
1 or 2	36 (94.7%)	151 (91.5%)	
3	2 (5.3%)	14 (8.5%)	
Diabetes Mellitus (%)	14 (36.8%)	52 (32.1%)	0.567
Pancreatic cancer (%)	10 (26.3%)	85 (51.5%)	
Ampullary cancer (%)	6 (15.8%)	12 (7.3%)	
Distal bile duct cancer (%)	15 (39.5%)	25 (15.2%)	
Duodenal cancer (%)	2 (5.3%)	2 (1.2%)	
IPMN (%)	3 (7.9%)	23 (13.9%)	
Others (%)	2 (5.3%)	18 (10.9%)	
Preoperative biliary drainage (%)	29 (76.3%)	128 (77.6%)	0.833
HbA1c (%)	5.8 (3.8–9.8)	5.8 (4.3–11.9)	0.594
WBC (×10^3^/μL)	5.2 (3.0–11.7)	5.1 (2.6–11.8)	0.945
Hb (g/dL)	11.9 (9.0–16.2)	12.3 (8.6–16.6)	0.279
Plt (×10⁴/μL)	20.1 (10.1–38.5)	22.2 (8.0–49.7)	0.159
CRP (mg/dL)	1.54 (0.02–9.55)	0.09 (0.01–3.33)	<0.001
Total bilirubin (mg/dL)	1.11(0.3–4.0)	1.26 (0.2–10.0)	0.610
Albumin (g/dL)	3.4 (2.4–4.7)	3.8 (2.2–4.7)	0.001
CAR	0.48 (0.01–2.81)	0.02 (0.002–1.18)	<0.001

POPF: Postoperative pancreatic fistula; BMI: Body mass index; CRP ASA: American Society of Anesthesiologists; IPMN: Intraductal papillary mucinous neoplasm; CRP: C-reactive protein; CAR: CRP to Albumin ratio.

**Table 2:** Intra- and postoperative variables in patients with and without postoperative pancreatic fistula.

Intra- and Postoperative	POPF Group	Non-POPF Group	*p*-Value
Variables	(*N* = 38)	(*N* = 165)	
Operation time (min)	602 (361–1003)	538 (318–1045)	0.003
Estimated blood loss (mL)	1133 (155–3450)	756 (50–6375)	0.001
Blood transfusion (%)	14 (36.8)	43 (26.1)	0.182
Blumgart method (%)	65.8%	78.8%	0.089
Portal vein resection (%)	3 (7.9)	37 (22.4)	0.042
Soft pancreas (%)	31 (81.6)	105 (63.6)	0.033
POCs excluding POPF-related POCs			
CD grade over III (%)	3 (7.9)	16 (9.7)	0.121

POCs, postoperative complications; POPF: postoperative pancreatic fistula; POPF-related POCs: intraabdominal bleeding, surgical site infection.

**Table 3:** Univariate and multivariate logistic regression analyses of predictors of postoperative pancreatic fistula. Univariate and multivariate analysis by logistical regression.

	Univariate		Multivariate	
Parameters	Odds Ratio (95% CI)	*p*-Value	Odds Ratio (95% CI)	*p*-Value
BMI ≥ 21.6	2.700 (1.233–5.913)	0.013	2.269 (0.757–6.801)	0.143
Estimated blood loss ≥ 790 (g)	4.391 (1.900–10.150)	0.001	2.980 (0.942–9.430)	0.063
Operation time ≧ 534 (min)	6.364 (2.368–17.108)	<0.001	4.715 (1.307–17.011)	0.018
Portal vein resection (%)	0.297 (0.086–1.019)	0.054		
Soft pancreas texture (%)	1.794 (0.796–4.043)	0.159		
CAR ≥ 0.09	32.928 (12.396–87.464)	<0.001	34.511 (11.748–101.381)	<0.001

CI, confidence interval; BMI, body mass index; CAR, C-reactive protein-to-albumin ratio.

## Data Availability

The data presented in this study are available in this article.
